# A rare pediatric case: Budd-Chiari Syndrome and upper gastrointestinal bleeding in a 5-year-old boy

**DOI:** 10.1016/j.ijscr.2025.110992

**Published:** 2025-01-29

**Authors:** Mohamed Ismail Ibrahim, Ahmed Abdi Aw Egge, Omar Ali Elmi, Mohamoud Hashi Abdi, Mohamed Ali Abdilahi, Abdirahman Omer Ali

**Affiliations:** a College of Health Sciences, School of Medicine and Surgery, Amoud University, Borama, Somalia; b School of Postgraduate Studies and Research, Amoud University, Amoud Valley, Borama 25263, Somalia; c Mass CTH, Pediatric department, Somalia; d Borama Regional Hospital, Radiology Department, Somalia

**Keywords:** Budd-Chiari syndrome, Hepatic venous outflow obstruction, Ascites, Hematemesis, Resource-limited settings, Transjugular intrahepatic portosystemic shunt (TIPS), Case report

## Abstract

**Introduction and importance:**

Budd-Chiari Syndrome (BCS) is a rare condition characterized by hepatic venous outflow obstruction, often presenting with ascites, hepatomegaly, and abdominal pain. The diagnosis and management of BCS, especially in resource-limited settings, pose significant challenges that can lead to poor patient outcomes.

**Case presentation:**

This case report describes a previously healthy individual presenting with progressive abdominal distention, hematemesis, and right upper quadrant pain. The patient had a history of trauma and underwent subsequent percutaneous drainage for ascites. Imaging studies revealed non-opacified hepatic veins, splenomegaly, ascites, and a “nutmeg liver” pattern, confirming the diagnosis of BCS. The patient received blood transfusions, diuretics, and nutritional support. A Transjugular Intrahepatic Portosystemic Shunt (TIPS) procedure was planned but ultimately hindered by financial constraints.

**Clinical discussion:**

This case highlights the challenges of managing BCS in resource-limited settings, particularly regarding access to specialized treatments like TIPS. It emphasizes the need for early diagnosis, multidisciplinary care, and the development of cost-effective treatment strategies to improve patient outcomes.

**Conclusion:**

The management of Budd-Chiari Syndrome is complex, particularly in settings with limited resources. This case underscores the importance of timely intervention and the need for innovative approaches to healthcare delivery that can accommodate financial barriers while ensuring patient safety and care quality.

## Highlights

• **Rare Case Presentation:** The case report describes a patient with Budd-Chiari Syndrome (BCS), a rare condition affecting hepatic venous outflow. This case underscores the importance of recognizing this rare entity in clinical practice.
• **Challenges of Diagnosis:** The patient's initial presentation was atypical, highlighting the diagnostic challenges associated with BCS, especially in resource-limited settings.
• **Impact of Resource Limitations:** The case highlights the significant impact of financial constraints on accessing essential treatments like TIPS, emphasizing the need for improved healthcare access in resource-limited environments.
• **Multidisciplinary Approach:** The patient's care required a multidisciplinary approach, involving various specialists and interventions. This underscores the importance of collaboration in managing complex medical conditions.
• **Importance of Early Intervention:** The patient's recurrent complications highlight the significance of early diagnosis and timely interventions for optimal outcomes in BCS management.

## 1 Introduction

Budd-Chiari syndrome (BCS) is a rare condition caused by either thrombotic or non-thrombotic obstruction of hepatic venous outflow. This condition is characterized by symptoms such as ascites, hepatomegaly, and abdominal pain. The estimated incidence of BCS is approximately 1 in 100,000 individuals in the general population, with fewer than 300 cases documented across three tertiary referral centers located in France, the United States, and the Netherlands [1]. The obstruction of hepatic venous outflow is the primary pathological factor in BCS, which typically manifests with a classic triad of abdominal pain, ascites, and hepatomegaly [2]. BCS can be classified as either primary or secondary; primary BCS arises from thrombosis, while secondary BCS is due to obstruction from tumor invasion or compression [3]. In Western countries, primary BCS is regarded as a rare hepatic manifestation linked to an underlying prothrombotic condition [4]. Often, this prothrombotic condition remains undiagnosed at the time the hepatic venous outflow obstruction becomes evident. Data from multiple centers indicate that between 25 % and 46 % of individuals with BCS have multiple concurrent prothrombotic disorders [5]. The primary form of BCS is exceedingly rare, with an estimated prevalence of 1 case per 1 million individuals annually, which is significantly below the threshold of 2 cases per 10,000 that qualifies as a rare disease [6]. Notably, this case highlights the challenges of diagnosing and managing BCS in a pediatric patient, particularly within resource-limited settings, and a unique presentation with history of trauma. The patient's unique presentation, including a history of trauma and subsequent complications, underscores the multifactorial nature of BCS and the urgent need for tailored management strategies. This case report aims to contribute to the discussion on the management of BCS in resource limited settings with emphasis on a non-classic presentation. This case is written in line with the SCARE 2023 guideline [7].

## 2 Case presentation

A 5-year-old male patient presented with a five-month history of progressive abdominal distention, the patient presented with four days of hematemesis, vomiting, diarrhea, right upper quadrant pain, and poor oral intake. One month prior to this presentation, the patient sustained a significant blunt abdominal trauma during a fall. The specific mechanism of injury such as the height of the fall was unavailable, however, the trauma required a 19-day hospitalization at a local health center. During this hospitalization, percutaneous drainage of ascites was performed, and diuretics were initiated look Fig. 3. The patient did not have umbilical catheterization during any of his medical procedures. Notably, the patient experienced dark urine, which was exacerbated by unknown factors but relieved with unspecified anti-diarrheal medication. There were no reports of jaundice, pale stools, or nausea. The patient had no known chronic diseases, previous hospitalizations, blood transfusions, or surgeries. There was no family history of similar conditions or autoimmune diseases. The patient reported no known drug allergies and was currently on Lasix and spironolactone.

**Fig. 1:**
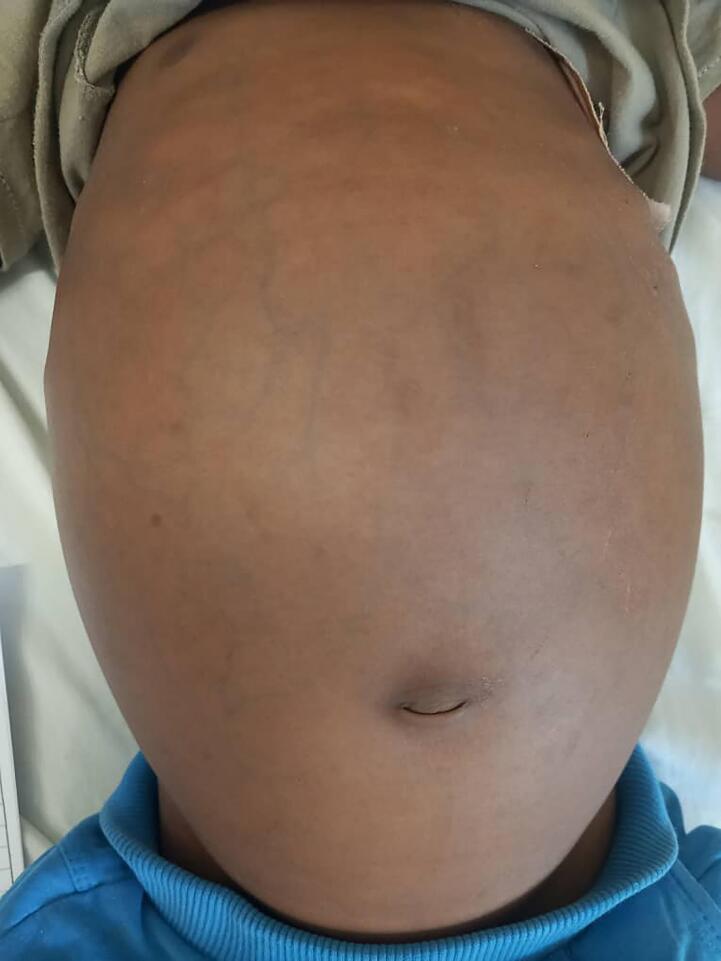
The image appears to show a person's abdomen, which is notably distended.

On examination, the patient displayed: Facial pallor, Massive abdominal ascites with a dull percussion note and no tenderness, Dilated visible veins in the abdomen, Hepatomegaly, with the liver edge palpable 3 cm below the right. A CT costal margin, Inverted umbilicus, a sign commonly associated with prolonged ascites look Fig. 1.

**Fig. 2:**
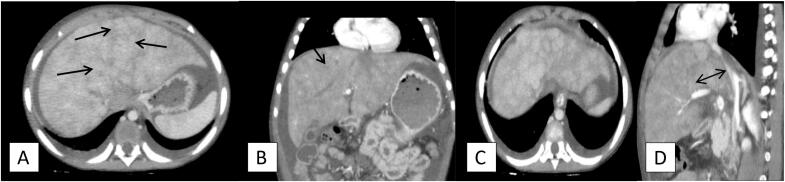
CT findings: (A, B) Axial and coronal CT post contrast image shows non-opacified hepatic veins (black arrows in the image A and B) with mild splenomegaly and moderate ascites C) axial post contrast image: Mildly enlarged liver with heterogeneous hypo attenuation and patchy enhancement (nutmeg liver) with minimal bilateral pleural effusion. d) Sagittal post contrast images: focal intrahepatic IVC narrowing (double headed arrow on D).

The ultrasound findings indicated non-opacified hepatic veins, raising suspicion for Budd-Chiari syndrome, along with mild splenomegaly suggestive of portal hypertension and moderate ascites linked to liver disease. A contrast-enhanced computed tomography (CECT) scan confirmed these findings, showing non-opacified hepatic veins and mild splenomegaly, consistent with portal hypertension. The CECT also revealed heterogeneous hypoattenuation and patchy enhancement of the liver, which is indicative of “nutmeg liver.” This finding is due to the characteristic pattern of sinusoidal congestion and hepatocyte necrosis resulting from impaired hepatic venous outflow. The liver showed an appearance of alternating areas of high and low attenuation, reflecting the centrilobular congestion and periportal sparing which contribute to the “nutmeg” appearance. The patchy enhancement seen was due to the inconsistent perfusion within the liver parenchyma. as well as focal intrahepatic narrowing of the inferior vena cava (IVC), suggesting vascular obstruction. These imaging results collectively confirm the diagnosis of Budd-Chiari syndrome look Fig. 2*.*

**Fig. 3:**
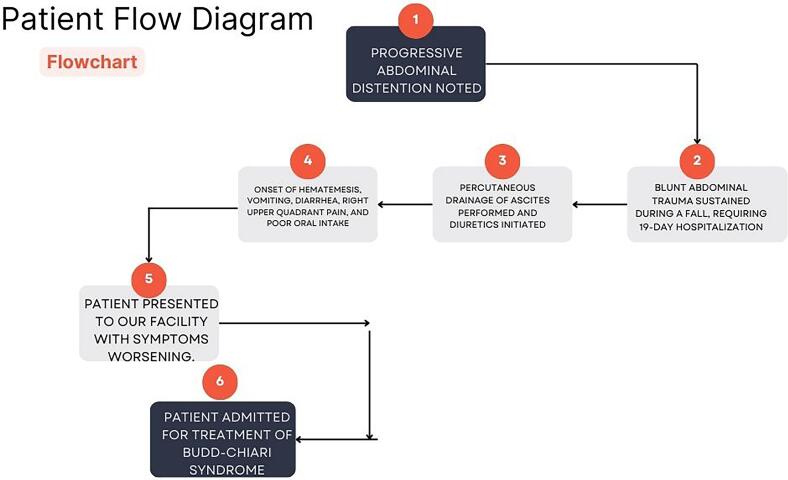
Patient flow chart.

The Complete Blood Count (CBC) indicated the presence of anemia, likely attributed to blood loss. Additionally, Liver Function Tests (LFTs) revealed elevations in transaminases, specifically aspartate aminotransferase (AST) and alanine aminotransferase (ALT), along with increased bilirubin levels, which could be correlated with liver congestion. Furthermore, Coagulation Studies demonstrated a potential prolongation of prothrombin time (PT) and partial thromboplastin time (PTT), reflective of compromised liver function. The specific lab results are as follows: Hemoglobin 8.1 g/dL (reference range: 11–13 g/dL), White Blood Cell count 7.5 × 10^9/L (reference range 4.5–11 × 10^9/L), Platelet count 230 × 10^9/L (reference range 150–400 × 10^9/L), AST 145 U/L (reference range: <40 U/L), ALT 102 U/L (reference range: <40 U/L), total bilirubin 3.4 mg/dL (reference range: 0.3–1.2 mg/dL), direct bilirubin 2.8 mg/dL(reference range < 0.3 mg/dL), Prothrombin time (PT) 18 s (reference range 11–13 s), partial thromboplastin time (PTT) 35 s (reference range 25–35 s), and INR 1.4 (reference range 0.8–1.2).

## 3 Diagnosis

The diagnosis of Budd-Chiari syndrome was established based on clinical findings, imaging studies, and laboratory results.

## 4 Treatment plan

Blood transfusions were given to correct anemia and stabilize hemodynamics. For fluid Management we utilized diuretics (Lasix, spironolactone) to reduce ascites. The specific doses of Lasix and Spironolactone at the initial treatment were 2 mg/kg/day and 1 mg/kg/day respectively and was later adjusted based on daily fluid balance and weight measurements. Nutritional Support: A high-calorie, high-protein diet was recommended, with possible enteral or parenteral nutrition to ensure adequate intake. Since then, initiation of anticoagulation therapy with warfarin to prevent further thrombosis and administration of liver protectants, such as silymarin, to safeguard liver cells. For Surgical Consultation: A surgical intervention for a Transjugular Intrahepatic Portosystemic Shunt (TIPS) was planned to alleviate portal hypertension by connecting the portal vein to a hepatic vein; however, financial constraints impeded the procedure. Detailed information regarding the cost of TIPS was not available.

## 5 Outcome and follow-up

Following discharge, the patient experienced a concerning relapse marked by recurrent hematemesis and epistaxis, necessitating readmission to Mas Hospital. This clinical deterioration prompted a comprehensive follow-up protocol designed for meticulous monitoring and intervention. The patient's vital signs (blood pressure, heart rate, respiratory rate, and temperature) were diligently assessed at regular intervals to detect any hemodynamic instability. Serial hematocrit and coagulation parameters were closely tracked to evaluate and manage any ongoing blood loss or coagulopathic tendencies. Liver function was serially assessed through liver function tests (LFTs) to monitor treatment efficacy and the status of hepatic health. Furthermore, the severity of ascites and changes in liver size were evaluated through regular abdominal ultrasound and computed tomography (CT) scans. Recognizing the importance of holistic care, the patient also received ongoing nutritional assessments and psychosocial support throughout the recovery process. The patient was monitored with biweekly follow up for the first month, then monthly follow up after stabilization of the symptoms. The follow-up protocol is in place for 6 months with possible extension of the follow-up protocol based on assessment of his status at the 6-month follow up.

## 6 Discussion

Budd-Chiari Syndrome (BCS) is a rare and potentially life-threatening condition that affects hepatic venous outflow, leading to significant morbidity and mortality. This case report highlights the clinical presentation, diagnostic approach, and challenges associated with managing BCS in a resource-limited setting. The patient presented with classic symptoms of BCS, including abdominal distention, hematemesis, and hepatomegaly. Imaging studies confirmed the diagnosis, revealing non-opacified hepatic veins, splenomegaly, ascites, and a “nutmeg liver” pattern. However, unlike classic presentations of BCS, our patient had an acute onset of symptoms after a prior abdominal trauma event and the symptoms were preceded by only abdominal distention. This is important because BCS may not be an immediate diagnosis and a thorough history including prior events is required.

Our findings are consistent with previous studies highlighting the diverse spectrum of BCS presentations. While some individuals may present with acute onset of symptoms, as seen in this case, others may experience a more insidious progression of the disease [1] [2]. The underlying etiology of BCS is often multifactorial, with an underlying prothrombotic condition identified in approximately 75 % of patients [6]. In our case, we investigated possible underlying prothrombotic conditions such as Protein C deficiency, Protein S deficiency, Anti-thrombin deficiency, Factor V Leiden and Prothrombin G20210A mutations. All these came back negative. This finding highlights the fact that not all BCS cases have an underlying prothrombotic condition. This case did not reveal any specific identifiable underlying prothrombotic condition, which further emphasizes the diagnostic challenge in identifying the cause of BCS in some patients.

The diagnostic workup for BCS requires a comprehensive approach incorporating clinical assessment, laboratory investigations, and imaging studies. The classic clinical triad of abdominal pain, ascites, and hepatomegaly, along with the characteristic imaging findings of hepatic vein obstruction and collateral formation, provides strong evidence for the diagnosis. However, the presence of these features can also be seen in other vascular liver diseases, such as Porto sinusoidal vascular disease, necessitating careful differentiation [6].

Treatment for BCS is multidisciplinary and involves addressing the underlying cause, managing complications, and preventing further thrombosis. Anticoagulation therapy is a cornerstone of management, although randomized trials are lacking [3]. Other therapeutic options include recanalization strategies, surgical shunting, TIPS, and ultimately, OLT. The selection of treatment depends on the severity of liver dysfunction, portal hypertension, and IVC obstruction [5] [8,9]. Our case underscores the critical role of timely intervention. While our patient stabilized with medical management, access to TIPS would have likely improved his outcome. Unfortunately, due to financial limitations this was not an option. However, the lack of availability of TIPS in many resource-limited settings necessitates the need for alternative management strategies. Some studies have highlighted the use of modified anticoagulation regimens with low-dose warfarin as a more affordable alternative [10,11]. These studies have found this to be effective in long term management and prevention of recurrence in patients with BCS [12]. In situations where TIPS is not available or contraindicated, less invasive local venous recanalization techniques can be utilized, although success rates vary [[13], [14], [15]]. Another potential option for management of patients in resource-limited areas is the integration of community-based support and long-term surveillance strategies. It has been noted that community-based healthcare providers can monitor patients and refer them to specialized centers when necessary [[16], [17], [18]]. Furthermore, reduction in cost of diagnostic tools can be achieved by exploring lower cost imaging techniques with comparable diagnostic accuracy to CT. This would allow more patients to undergo early testing and management of the condition [[19], [20], [21]]. We acknowledge that these are not replacements for interventions such as TIPS but may aid in increasing patient access to care.

A recent meta-analysis reported that the median 1-, 5-, and 10-year survival rates of patients receiving interventional radiological treatments were 93 %, 83 %, 73 %, respectively. The survival rates of patients receiving surgery other than LT were 81 %, 75 %, 72.5 %, respectively, and those of patients receiving LT were 82.5 %, 70.2 %, 66.5 %, respectively. The 1- and 5-year survival rates of patients receiving medical therapy were 68.1 % and 44.4 %, respectively [22] [23].

The patient in this case experienced a complex clinical course, marked by recurrent hematemesis and epistaxis, highlighting the need for close monitoring and prompt management of complications. While the patient was initially scheduled for a TIPS procedure, financial constraints prevented the implementation of this vital intervention. This underscores the significant impact of resource limitations on the management of complex conditions, particularly in resource-limited settings. Interventional radiology, particularly Transjugular Intrahepatic Portosystemic Shunt (TIPS), plays a crucial role in the management of Budd-Chiari Syndrome (BCS), and can be a life-saving procedure in the management of this condition [24]. The case emphasizes the need for innovative and cost-effective strategies for the management of BCS and similar conditions in resource limited areas.

Despite the challenges, our case report emphasizes the importance of early diagnosis and timely intervention in improving outcomes for patients with BCS. The development of strong strategies for managing this rare condition within the constraints of resource-limited healthcare systems is essential.

This case report contributes to the growing body of knowledge on the diagnosis and management of BCS. It highlights the importance of a multidisciplinary approach, the need to address underlying prothrombotic conditions, and the challenges faced in resource-limited settings. It also stresses the need for more cost-effective strategies for diagnosis and management in resource limited settings.

## 7 Contextualization with Sustainable Development Goals (SDGs)

This case report aligns with several SDGs, including:

• **SDG 3: Good Health and Well-being:** Early detection and management of rare conditions like BCS are crucial for improving health outcomes and reducing morbidity and mortality.
• **SDG 10: Reduced Inequalities:** Access to high-quality healthcare, including specialized treatments for rare diseases, is essential to ensure equity and reduce health disparities.
• **SDG 17: Partnerships for the Goals:** Effective management of BCS requires collaboration between healthcare providers, researchers, and policymakers to improve diagnosis, treatment, and access to care.

## 8 Strengths and weaknesses

• This case report provides valuable insights into the challenges of managing Budd-Chiari Syndrome (BCS) in a resource-limited setting, yet it is important to recognize both its strengths and limitations.

## 9 Strengths

• Detailed clinical presentation: This case report provides a detailed clinical presentation of a rare condition (BCS) in a pediatric patient within a resource-limited environment. It outlines the history of the presentation, and its evolution and progression.
• Comprehensive diagnostic process: The manuscript outlines a step-by-step diagnostic approach, incorporating clinical evaluation, laboratory findings, and imaging studies, highlighting key diagnostic features such as the “nutmeg liver” appearance and non-opacified hepatic veins.
• Discussion of management challenges: The report discusses in detail the significant constraints imposed by financial limitations on treatment options and highlights the difficulties faced in accessing essential interventional procedures like TIPS. Furthermore, the manuscript highlights the need for development of alternative treatment strategies in these settings.
• Highlighting a unique presentation: The case report emphasizes the unique presentation of BCS following a traumatic event, an unusual aspect not typically documented in classic presentations of the syndrome.
• Focus on resource-limited setting: The manuscript addresses a critical gap in the literature by focusing on the management of BCS in a setting with limited resources, highlighting the ethical and practical considerations involved.

## 10 Weaknesses

• Lack of specific information: While we have detailed the blunt nature of the trauma, we acknowledge that specifics of the event (exact mechanism, height of fall) were unavailable in the patient's records.
• Limited follow-up duration: The follow-up period for this case is limited, and we acknowledge that longer follow-up may provide further insight into long term outcomes.
• Lack of definitive outcome: Although the patient improved after the initial management, the lack of access to TIPS meant the patients long term outcome remains uncertain.
• Single case report: As a single case report, the findings may not be generalizable to all patients with BCS, and further studies are required.
• Lack of advanced interventions: The constraints of the resource limited setting resulted in the patient not having access to advanced interventional procedures such as TIPS, and thus limits the evaluation of outcomes for this patient and the disease process.
• Limited access to investigations: The lack of access to more advanced diagnostic tests, such as liver biopsy and genetic studies, limits our ability to fully elucidate the underlying cause of the patient's BCS.

## 11 Conclusion

This case report highlights the diagnostic and therapeutic challenges of managing Budd-Chiari syndrome (BCS) in a resource-limited setting. The patient's presentation, confirmed by imaging, underscored the importance of early diagnosis and timely intervention for optimal outcomes. However, the case also emphasizes the significant impact of financial constraints on accessing vital interventions like TIPS, highlighting the need for improved access to healthcare in resource-limited settings. Furthermore, the unique presentation of this case, with prior trauma and delayed acute presentation of hematemesis, adds to the literature. Further research and development of cost-effective treatment strategies for BCS are essential, emphasizing the need for collaborative efforts to improve care for patients with rare conditions. The case also stresses the need for thorough history taking in order to determine risk factors for this rare disease.

Consent for publication statement.

We have obtained written consent from the patient's legal guardian to publish this case report, including any associated images or clinical data. The guardian has been informed of the purpose of the publication, its potential risks and benefits, and the steps taken to ensure patient confidentiality. The patient's identity has been protected, and all identifying information has been removed or altered to maintain anonymity. We acknowledge that this case report holds educational and scientific value, and we grant permission for its publication in a reputable medical journal or platform.

## Consent

Written informed consent was obtained from the patient's parents/legal guardian for publication and any accompanying images. A copy of the written consent is available for review by the Editor-in-Chief of this journal on request.

## Ethical approval

This case report is exempt from ethnical approval.

## Guarantor

Abdirahman Omer Ali.

## Ethical considerations

The study protocol, case investigation, and consent form were thoroughly examined by the institutional review board of the College of Health Sciences at Amoud University. They granted approval for the study, along with the Ministry of Health and Mass Hospital in Awdal Region, Somaliland (MCTH-150/2024). Prior to participation, written informed consent was obtained from every individual involved.

## Funding

The study did not receive funding.

## Author contribution

Dr. Mohamed Ismail Ibrahim, Dr. Omar Ali Elmi, Dr. Ahmed Abdi Aw Egge, Dr. abdirahman Omer ali and Dr. Mohamed individuals contributed to taking history and providing care to the patient throughout her hospital stay. Additionally, Dr. abdirahman Omer Ali contributed to the development of the manuscript. Dr. Mohamoud Hashi Abdi is the radiologist.

## Declaration of competing interest

The authors affirm that there are no conflicts of interest pertaining to the publication of this article.
